# Human Placental Genomic Instability Predicts Adverse Pregnancy Outcomes

**DOI:** 10.21203/rs.3.rs-7419733/v1

**Published:** 2025-09-08

**Authors:** Molly Huang, Cindy Barba, Hector J. Chavez, Avery S. Williams, Nicholas Guo, Rhea Robertson, Emily Zhang, Arundhati Calambur, Cayla Mason, Katharine K. Nelson, Morgan Meads, Omonigho Aisagbonhi, Mariko Horii, Kathleen M. Fisch

**Affiliations:** 1 Department of Obstetrics, Gynecology & Reproductive Sciences, University of California, San Diego, La Jolla, CA, USA.; 2 Bioinformatics and Systems Biology Graduate Program, University of California, San Diego, La Jolla, CA, USA.; 3 Center for Perinatal Discovery, University of California, San Diego, La Jolla, CA, USA.; 4 Department of Pathology, University of California, San Diego, La Jolla, CA, USA.; 5 Biomedical Sciences Graduate Program, University of California, San Diego, La Jolla, CA, USA.; 6 Center for Computational Biology & Bioinformatics, University of California, San Diego, La Jolla, CA, USA.

## Abstract

Preeclampsia is a leading cause of pregnancy-related death, accounting for over 50,000 maternal and 500,000 fetal deaths worldwide each year^1–4^. Preeclampsia has been linked to confined placental mosaicism, which underscores a potential role of placental genomic instability in driving adverse pregnancy outcomes. Here, using bulk RNA sequencing from 59 preeclamptic and 53 normotensive pregnancies, we explored somatic genomic instability and hypoxia with respect to clinical maternal-placental-neonatal outcomes. We found that genomic instability increased the probability of delivering at an earlier gestational age with a diagnosis of preeclampsia, maternal vascular malperfusion placental lesions, and small for gestational age neonates. Notably, genomic instability and hypoxia are predictive biomarkers for all three adverse pregnancy outcomes. In an induced pluripotent stem cell-derived trophoblast stem cell model, we observed increased genomic instability in trophoblast stem cells obtained from placentas demonstrating maternal vascular malperfusion with preeclampsia. Additionally, increased genomic instability correlated with reduced extravillous trophoblast invasion, implicating a functional role for genomic instability. These findings provide promising insights into the underlying mechanisms of genomic instability in the placenta which may be useful biomarkers for early clinical diagnosis of placental injury underlying preeclampsia.

## Introduction

Preeclampsia (PE) is a hypertensive disorder of pregnancy that affects 3–8% of pregnancies and is responsible for 10–15% of maternal mortality^5^. A hallmark of PE is placental dysfunction, which occurs when the placenta fails to meet the nutrient and oxygen demands of the fetus for normal development and growth, leading to small for gestational age (SGA) neonates^6^. Incomplete maternal vascular remodeling early in gestation is a common finding in placental dysfunction that leads to a pattern of placental injury termed maternal vascular malperfusion (MVM)^7^. Patterns of placental injury can be identified by pathologic examination of this organ after delivery and diagnosed based on specific gross and histopathologic findings^8^. MVM is the most common placental injury pattern observed in early onset PE^9^, and is defined by the presence of at least two of the following: small placental disc, decidual arteriopathy, accelerated villous maturation, villous infarction, and hematomas^10^. Clinically, placental injury can only be identified after delivery, despite the fact that PE has a long subclinical phase that would benefit from early clinical intervention^11^, which motivates biomarker discovery to identify placental injury *in utero*^12^.

Interestingly, genomic instability characterized by high somatic mutational burden is a normal feature of development in the placenta^13^, which is hypothesized to result from the rapid growth and proliferation of villous trophoblasts, the main epithelial cells of the placenta, paired with limited DNA repair mechanisms during early gestation^14^. While genomic instability in healthy placental tissue has been identified, confined placental mosaicism, when chromosomal abnormalities exist within the placenta but not the fetus, has been associated with PE and low birthweight^15^. This highlights the potential clinical relevance of genomic instability in disease pathogenesis in the placenta.

Here, we report that placental genomic instability predicts PE, MVM, and SGA neonates. We evaluated bulk RNA sequencing (RNA-seq) from PE and normotensive placentas for genomic instability, mutational signatures, and rare damaging mutations. To uncover the functional roles of increased genomic instability, we evaluated an induced pluripotent stem cell (iPSC)-derived trophoblast stem cell (TSC) model, finding increased genomic instability in TSCs with PE with MVM relative to normotensive without MVM. Furthermore, we discovered increased genomic instability correlated with reduced extravillous trophoblast invasion. Together, these findings provide promising insights into the clinical and functional consequences of placental genomic instability and hypoxia score and their potential utility as circulating biomarkers for early clinical diagnosis of PE with MVM.

## Results

### Discovery cohort characteristics.

**Extended Data Fig. 1:**
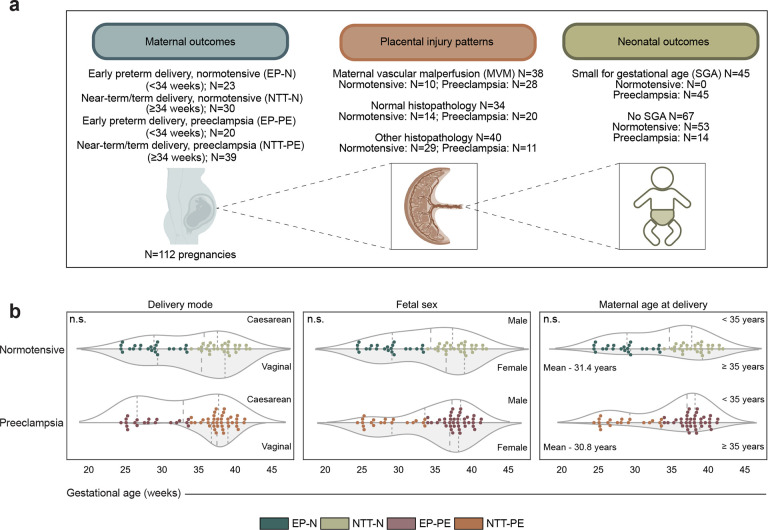
Additional clinical demographics of placentas in the discovery cohort and computational analysis workflow. **a,** Overview of the sample sizes within the clinical outcomes of the discovery cohort. Created in BioRender. Fisch, K. (2026) https://BioRender.com/rt24fbt. **b,** Matched clinical outcomes stratified by maternal outcome across gestational age at delivery. Distribution of mode of delivery, fetal sex, and maternal age at delivery. n.s., not significant; EP-N, early preterm delivery, normotensive; NTT-N, near-term/term delivery, normotensive; EP-PE, early preterm delivery, preeclampsia; NTT-PE, near-term/term delivery, preeclampsia. *P* values from a Chi-squared test.

**Fig. 1:**
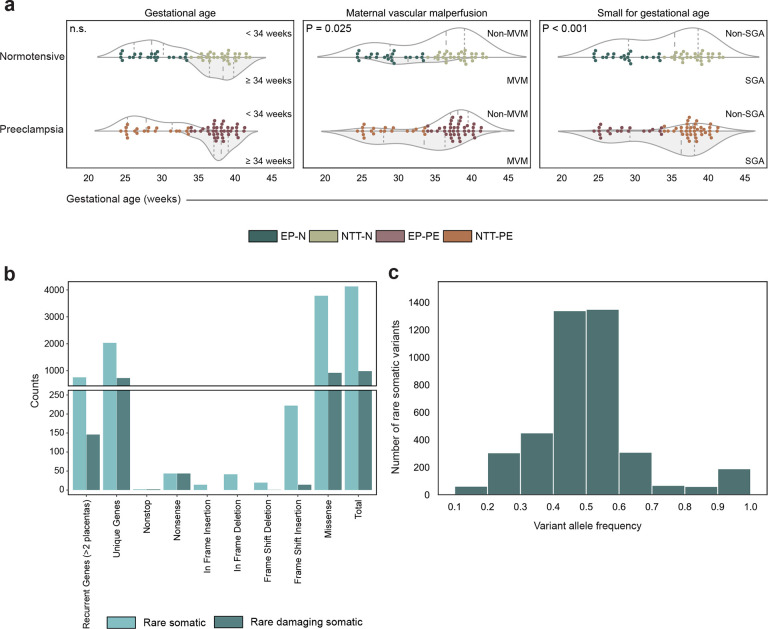
Clinical demographics of the placentas in the discovery cohort. **a,** Clinical outcomes stratified by maternal outcome across gestational age at delivery. Distribution of maternal age at delivery, maternal vascular malperfusion, and neonatal outcomes. n.s., not significant; MVM, maternal vascular malperfusion; SGA, small for gestational age; EP-N, early preterm delivery, normotensive; NTT-N, near-term/term delivery, normotensive; EP-PE, early preterm delivery, preeclampsia; NTT-PE, near-term/term delivery, preeclampsia. *P* values from a Chi-squared test. **b,** Counts of genes and somatic variants classified as rare and rare damaging. **c,** Counts of variant allele frequency of rare somatic variants.

Bulk RNA sequencing (RNA-seq) was performed on a discovery cohort of 112 human placentas. Early preterm (EP) delivery is defined as a gestational age at delivery of less than 34 weeks^16^ and is of interest because infants born prior to 34 weeks have an increased risk of neonatal mortality and morbidity^17^. Additionally, preeclampsia (PE) with delivery before 34 weeks is considered a distinct clinical subtype of PE^18^. We first classified these placentas by maternal hypertensive outcomes: 23 early preterm delivery normotensive (EP-N), 30 near-term/term delivery normotensive (NTT-N), 20 early preterm delivery with preeclampsia (EP-PE), and 39 near-term/term delivery with preeclampsia (NTT-PE) (Extended Data Fig. 1a). Placentas stratified by PE were matched for gestational age (P.adj = 1.000, two sample T-test; Fig. 1a, Supplementary Table 1), mode of delivery (P.adj = 1.000, Chi-squared test; Extended Data Fig. 1b, Supplementary Table 1), fetal sex (P.adj = 1.000, Chi-squared test; Extended Data Fig. 1c, Supplementary Table 1), and maternal age at delivery (P.adj = 1.000, Chi-squared test; Extended Data Fig. 1d, Supplementary Table 1).

Placental injury patterns were determined through histopathological review. Maternal vascular malperfusion (MVM) was observed in 38 of the 112 placentas (Extended Data Fig. 1a), of which 10 were normotensive and 28 had PE. 15 of 20 EP-PE placentas had MVM. The presence of MVM was significantly higher in PE compared to normotensive placentas (P = 0.025, Chi-squared test; Fig. 1a, Supplementary Table 1).

Small for gestational age (SGA) neonates were only included in the PE cohort, with 45 of 59 PE pregnancies having SGA neonates (P < 0.001, Chi-squared test; Fig. 1a, Supplementary Table 1). 18 of 20 EP-PE placentas resulted in SGA neonates. Histopathology classification was associated with different rates of SGA, with 71.1% of MVM placentas versus 24.3% of normal histopathology placentas associated with SGA (P < 0.001, Fisher’s exact test; Supplementary Table 2). These findings suggest that vascular lesions, such as MVM, can be used as a proxy for placental dysfunction due to their association with SGA, regardless of maternal clinical disease.

To explore the mutational landscape of placental villous tissue across hypertensive disease states and patterns of placental injury, we investigated rare single-nucleotide and insertion/deletion somatic variants. Variants were filtered for somatic variants only^19^. Across all samples, 983 somatic variants were rare (AF < 0.01) and damaging within 731 unique genes, and 4,131 variants were rare within 2,036 unique genes (Fig. 1b, Supplementary Table 3). The variant allele frequency of placental variants ranged from 0.18 to 0.99 (Fig. 1c).

### Somatic mutational landscape of preeclampsia.

**Fig. 2:**
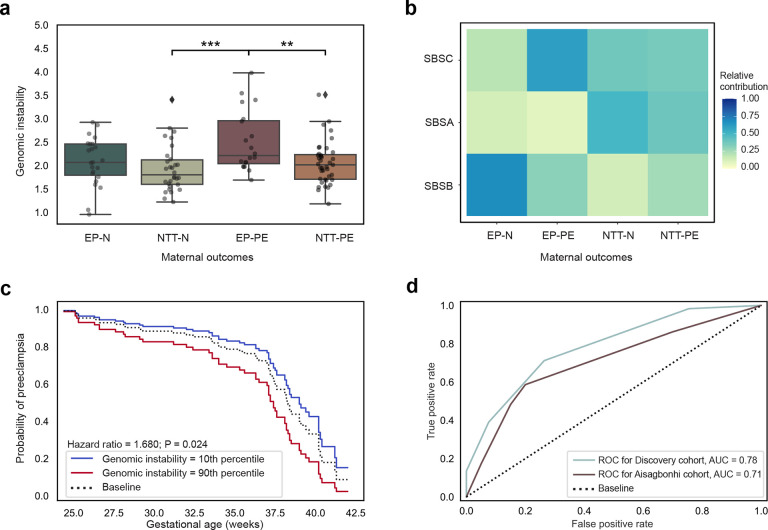
Genomic instability, oxidative stress mutational signatures, and hypoxia score are associated with preeclampsia. **a,** Comparison of the genomic instability between maternal outcomes. ***P<0.001, **P<0.01; *P* values from a two-sided Mann-Whitney U test with Benjamini-Hochberg correction. EP-N, early preterm delivery, normotensive; NTT-N, near-term/term delivery, normotensive; EP-PE, early preterm delivery, preeclampsia; NTT-PE, near-term/term delivery, preeclampsia. **b,** SBS mutational signatures by maternal outcomes. SBS, single base substitution. **c,** Probability of developing preeclampsia at each week of gestational age between placentas in the 10th percentile (blue) and 90th percentile (red) of genomic instability. *P* value from a Cox proportional hazards model. **d,** Receiver operator characteristic curve for K-Nearest Neighbors model predicting preeclampsia trained using genomic instability, hypoxia score, and fetal sex as features. Performance is quantified by area under receiver operator characteristic curve, noted per cohort. ROC, receiver operator characteristic curve; AUC, area under curve.

To determine the association of genomic instability with clinical outcomes, we defined genomic instability in each placenta by expressed mutational burden, which is the number of expressed rare somatic variants per capture region at 50X coverage per megabase^20^. We found that genomic instability was higher in EP-PE compared to NTT-N and NTT-PE (P < 0.01, two-sided Mann-Whitney U test with Benjamini-Hochberg correction; Fig. 2a), indicating a higher number of rare somatic variants in placentas delivered EP-PE.

**Extended Data Fig. 2:**
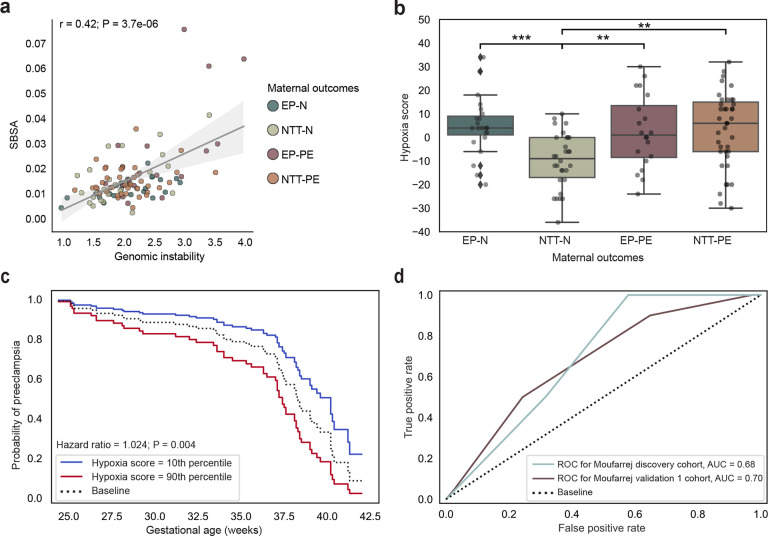
Genomic instability and hypoxia score are moderately predictive for preeclampsia in circulating RNA. **a,** Correlation of SBSA mutational signature contribution and genomic instability. *P* value from Spearman’s rank-order correlation test. EP-N, early preterm delivery, normotensive; NTT-N, near-term/term delivery, normotensive; EP-PE, early preterm delivery, preeclampsia; NTT-PE, near-term/term delivery, preeclampsia. **b,** Comparison of hypoxia score between maternal outcomes. ***P<0.001, **P<0.01; *P* value from a two-sided Mann-Whitney U test with Benjamini-Hochberg correction. **c,** Probability of developing preeclampsia at each week of gestational age between placentas in the 10th percentiles (blue) and 90th percentile (red) of hypoxia score. *P* value from a Cox proportional hazards model. **d,** Receiver operator characteristic curve for K-Nearest Neighbors model predicting preeclampsia trained using genomic instability and hypoxia score as features on the Moufarrej discovery cohort. Performance is quantified by area under receiver operator characteristic curve, noted per cohort. ROC, receiver operator characteristic curve; AUC, area under curve.

Next, we analyzed single base substitution (SBS) mutational signatures^21^ to explore mutational processes driving genomic instability in EP-PE. We identified contributions from SBSA (SBS1-like, a clock-like signature that introduces background mutations and correlates with age^22,23^), SBSB (SBS5-like, a common signature across human tissues possibly as a result of endogenous damage and repair^23^), and SBSC (SBS18-like, attributed to oxidative DNA damage^23^) (Fig. 2b). Genomic instability showed a significant positive correlation between the clock-like signature (SBSA) contribution and genomic instability (r = 0.42, P < 3.7e-06, Spearman’s rank-order correlation; Extended Data Fig. 2a). Both NTT-PE and NTT-N were characterized by higher levels of the oxidative DNA damage signature (SBSC) and the clock-like signature (SBSA). In contrast, the mutational signature of EP-N was primarily represented by the endogenous DNA damage and repair signature (SBSB) (Fig. 2b). The mutational signature of EP-PE was primarily represented by the oxidative DNA damage signature (SBSC), which is characterized by C>A substitutions and attributed to oxidative DNA damage, with a minor component of T>A substitutions (similar to SBS34) resulting from DNA polymerase slippage at adjacent adenine and thymine homopolymer tract boundaries^23^. This observation suggests that oxidative stress may be associated with placental genomic instability in placental villous tissue in EP-PE, leading us to profile placental response to hypoxia, represented by gene expression scoring using a hypoxia gene signature^24^. A higher placental hypoxia score was associated with EP-N, EP-PE, and NTT-PE compared to NTT-N (P < 0.01, two-sided Mann-Whitney U test with Benjamini-Hochberg correction; Extended Data Fig. 2b), demonstrating the relevance of a hypoxic environment to the pathogenesis of placental dysfunction.

### Molecular predictors of preeclampsia.

Predicting PE using molecular biomarkers has grown increasingly relevant of late due to the rise of non-invasive blood tests that can measure cell-free DNA and RNA^25,26^. As such, we profiled whether genomic instability and hypoxia score could be predictive biomarkers of clinical outcomes in placental tissue and circulating RNA. We found that higher levels of genomic instability (Hazard ratio = 1.680, P = 0.024, Cox proportional-hazards test; Fig. 2c) and hypoxia score (Hazard ratio = 1.024, P = 0.004, Cox proportional hazards model; Extended Data Fig. 2c) in placental RNA were significantly linked to an earlier onset of PE. Next, a K-Nearest Neighbors (KNN) model was trained using genomic instability, hypoxia score, and fetal sex as features in the discovery cohort and tested on the Aisagbonhi cohort^27^ (n = 20 normotensive, n = 29 PE; Supplementary Table 4), which both profiled placental RNA. The KNN model had predictive performance with an area under the curve (AUC) of 0.78 and accuracy of 72.3% on the discovery cohort and an AUC of 0.71 and an accuracy of 67.3% on the Aisagbonhi cohort (Fig. 2d).

We replicated the KNN model using circulating RNA in maternal circulation from Moufarrej et al.^26^ in pregnancies at greater than 23 weeks of gestational age using their discovery and validation 1 cohorts (Discovery: n = 37 normotensive, n = 20 PE; Validation 1: n = 19 normotensive, n = 6 PE; Supplementary Table 5) to determine if genomic instability and hypoxia score are relevant non-invasive biomarkers for predicting PE in maternal circulation. We identified rare variants to calculate genomic instability and calculated hypoxia score from gene expression using identical methods to those used for placental tissue. The KNN model had an AUC of 0.68 and accuracy of 66.7% on the Moufarrej discovery cohort and an AUC of 0.70 and an accuracy of 64.0% on the Moufarrej validation 1 cohort (Extended Data Fig. 2d). Together, these results suggest that genomic instability and hypoxia score have the potential to be relevant biomarkers for predicting PE.

### Predictors of maternal vascular malperfusion.

**Fig. 3:**
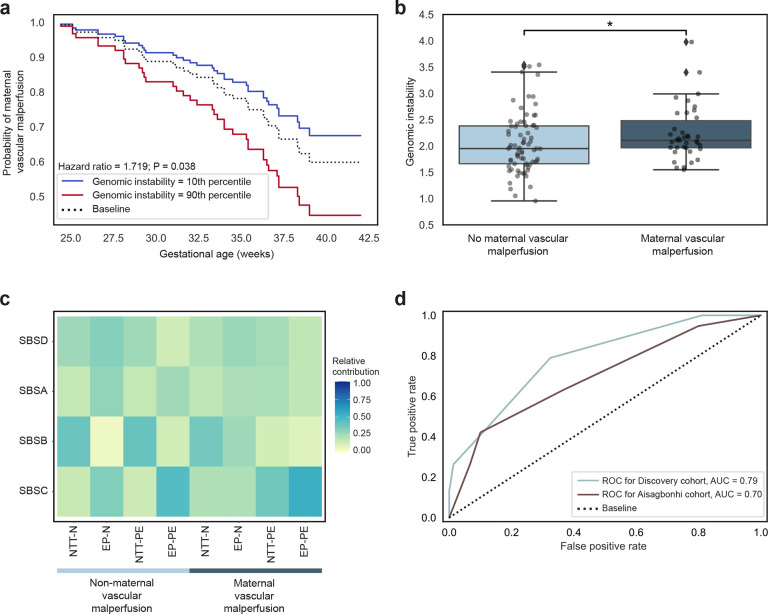
Genomic instability and hypoxia score are predictive for maternal vascular malperfusion. **a,** Probability of developing maternal vascular malperfusion at each week of gestational age between placentas in the 10th percentile (blue) and 90th percentile (red) of genomic instability. *P* value from a Cox proportional hazards model. **b,** Comparison of genomic instability between non-maternal vascular malperfusion and maternal vascular malperfusion samples. *P<0.05; *P* value from a one-sided Mann Whitney U test. **c,** SBS mutational signatures by maternal outcomes with and without maternal vascular malperfusion. SBS, single base substitution; EP-N, early preterm delivery, normotensive; NTT-N, near-term/term delivery, normotensive; EP-PE, early preterm delivery, preeclampsia; NTT-PE, near-term/term delivery, preeclampsia. **d,** Receiver operator characteristic curve for K-Nearest Neighbors model predicting maternal vascular malperfusion trained using genomic instability, hypoxia score, and fetal sex as features. Performance is quantified by area under receiver operator characteristic curve, noted per cohort. ROC, receiver operator characteristic curve; AUC, area under curve.

Due to the high frequency of MVM in PE placentas, we investigated the relationship between genomic instability and hypoxia score with MVM. Higher levels of genomic instability were linked to delivery at earlier gestational ages with MVM (Hazard ratio = 1.719, P = 0.038, Cox proportional-hazards model; Fig. 3a). Furthermore, genomic instability was higher in placentas with MVM regardless of maternal clinical outcome of PE (P = 0.021, one-sided Mann Whitney U test; Fig. 3b). We observed mutational signatures characterized by the clock-like signature (SBSA), the endogenous DNA damage and repair signature (SBSB), the oxidative DNA damage signature (SBSC), and a signature with unknown etiology (SBSD) in placentas with MVM and PE (Fig. 3c). MVM with PE was characterized by the oxidative DNA damage signature (SBSC) while MVM without PE was characterized by the endogenous DNA damage and repair signature (SBSB), revealing distinct molecular signatures associated with MVM (Fig. 3c).

Clinically, placental injury can only be identified after delivery, although PE has a long subclinical phase that would benefit from early clinical intervention which motivates the need for biomarkers capable of identifying placental injury in utero. Within placental tissue only, we trained a KNN model using genomic instability, hypoxia score, and fetal sex as features in the discovery cohort and tested on the Aisagbonhi cohort (n = 30 non-MVM, n = 19 MVM). The KNN model was predictive for MVM with an AUC of 0.78 and accuracy of 73.2% on the discovery cohort and an AUC of 0.71 and an accuracy of 71.4% on the validation cohort (Fig. 3d). This demonstrates the potential for genomic instability and hypoxia score as features for predicting MVM.

### Predictors of small for gestational age neonates.

**Fig. 4:**
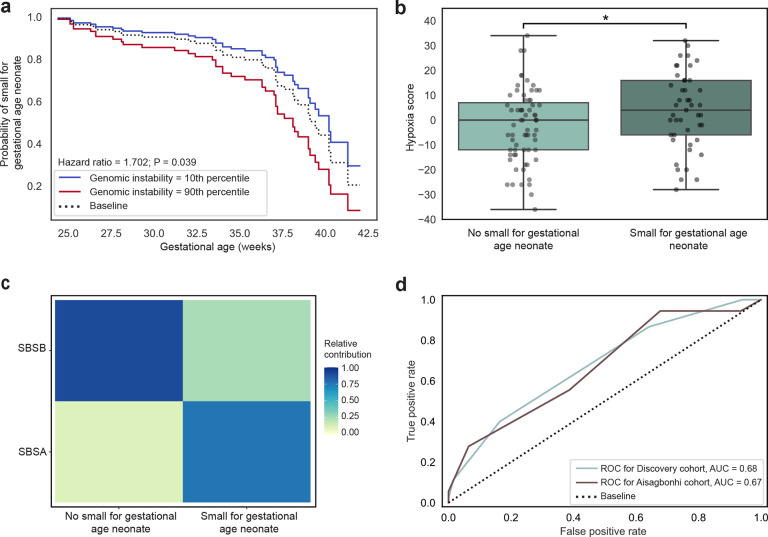
Placental hypoxia is associated with small for gestational age neonates. **a,** Probability of developing a small for gestational age neonate at each week of gestational age between placentas in the 10th quartile (blue) and 90th quartile (red) of hypoxia score. *P* value from a Cox proportional hazards model. **b,** Comparison of the hypoxia score between non-small for gestational age and small for gestational age. * *P* < 0.05; *P* value from a one-sided Mann-Whitney U test. **c,** SBS mutational signatures stratified by small for gestational age. SBS, single base substitution. **d,** Receiver operator characteristic curve for K-Nearest Neighbors model predicting small for gestational age neonate trained using genomic instability, hypoxia score, fetal sex, and maternal age at delivery as features. Performance is quantified by area under receiver operator characteristic curve, noted per cohort. ROC, receiver operator characteristic curve; AUC, area under curve.

We next set out to investigate whether placentas resulting in SGA neonates demonstrate similar genomic instability and mutational signatures. Again, higher levels of genomic instability were linked to delivery at earlier gestational ages with SGA neonates (Hazard ratio = 1.702, P = 0.039, Cox proportional-hazards model; Fig. 4a). SGA placentas had a higher hypoxia score compared to non-SGA placentas (P = 0.020, one-sided Mann-Whitney U test; Fig. 4b), demonstrating the relevance of a hypoxic environment to SGA. Non-SGA placentas were strongly characterized by the endogenous DNA damage and repair signature (SBSB) compared to SGA placentas, which were characterized by both the clock-like signature (SBSA) and the endogenous DNA damage and repair signature (SBSB), with a larger relative contribution of the clock-like signature (SBSA; Fig. 4c).

To test genomic instability and hypoxia score as biomarkers for SGA in placental tissue, we once more trained a KNN model using genomic instability, hypoxia score, maternal age at delivery, and fetal sex as features on the discovery cohort. The KNN model had limited predictive ability for SGA with an AUC of 0.68 and accuracy of 66.1% on the discovery cohort and an AUC of 0.67 and an accuracy of 59.2% on the Aisagbonhi cohort (n = 31 non-SGA, n = 18 SGA) (Fig. 4d).

### Genomic instability disrupts pathways important for placental health.

**Fig. 5:**
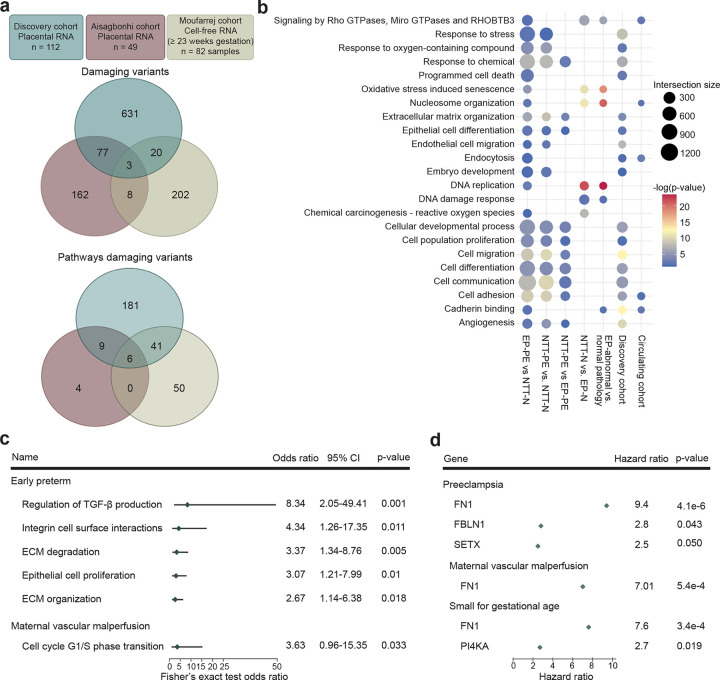
Damaging variants during placental dysfunction leads to dysregulated pathways. **a,** Overlap of damaging variants and the pathway of damaging variants called from the discovery, Aisagbonhi, and Moufarrej cohorts. **b,** Pathway enrichment comparisons of clinical outcomes and rare variant pathway enrichment in dysregulated pathways. EP-PE, early preterm delivery, preeclampsia; NTT-N, near-term/term delivery, normotensive; NTT-PE, near-term/term delivery, preeclampsia; EP-N, early preterm delivery, normotensive; EP, early preterm delivery. **c,** Fisher’s exact test odds ratio of pathways enriched in early preterm and maternal vascular malperfusion placentas. *P* value from a Fisher’s exact test. CI, confidence interval; ECM, extracellular matrix. **d,** Hazard ratio of a variant in each gene for preeclampsia, maternal vascular malperfusion, and small for gestational age neonates. *P* value from a Fisher’s exact test.

As genomic instability was correlated with PE, MVM and SGA, we compared rare damaging somatic variants in the discovery placental cohort (n=112) with the two independent cohorts for validating the predictive models: the Aisagbonhi placental cohort (n=49)^27^ and the Moufarrej circulating RNA cohort (n=82)^26^. We found that 80 of the 731 genes harboring rare damaging variants identified in the discovery cohort can be replicated in the independent Aisagbonhi validation cohort (Fig. 5a). Furthermore, 23 genes harboring rare damaging variants in placental villous tissue were also observed in the Moufarrej circulating cell-free RNA cohort (Fig. 5a). At the pathway level, numerous dysregulated pathways by gene expression were similarly enriched in the discovery and circulating cell-free RNA cohorts including programmed cell death, embryo development, and cell migration and adhesion, among other pathways biologically relevant to the etiology of PE (False discovery rate < 0.05, Fisher’s exact test; Fig. 5b, Supplementary Table 6–12). This further validates the results obtained from the discovery cohort.

**Extended Data Fig. 3:**
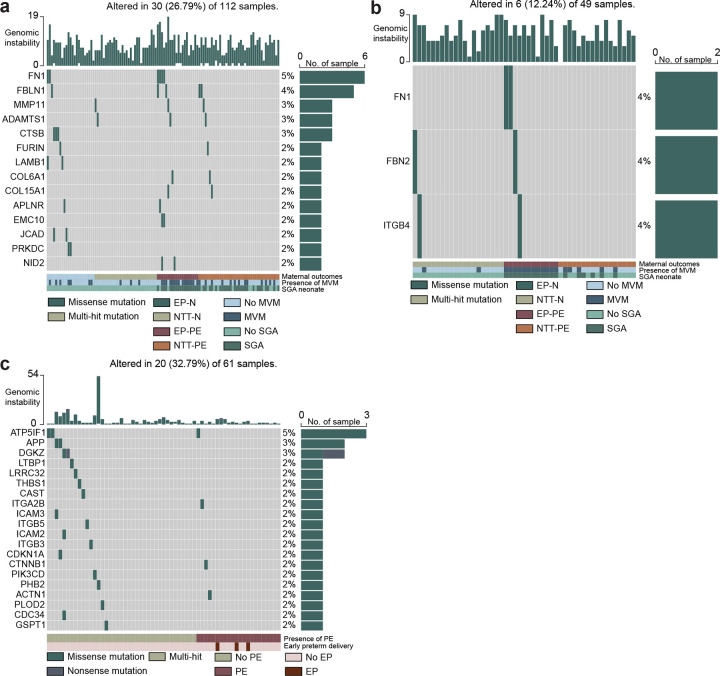
Recurrently mutated rare damaging variants in discovery, Aisagbonhi, and Moufarraj cohorts. **a,** Waterfall plot of genes recurrently mutated in significant pathways in the discovery cohort. EP, early preterm; NTT, near-term/term; NTT-N, near-term/term delivery, normotensive; EP-N, early preterm delivery, normotensive; NTT-PE, near-term/term delivery, preeclampsia; MVM, maternal vascular malperfusion; SGA, small for gestational age. **b,** Waterfall plot of genes recurrently mutated in significant pathways in the Aisagbonhi cohort. **c,** Waterfall plot of genes recurrently mutated in significant pathways in the Moufarraj cohort. PE, preeclampsia; EP, early preterm delivery.

**Extended Data Fig 4:**
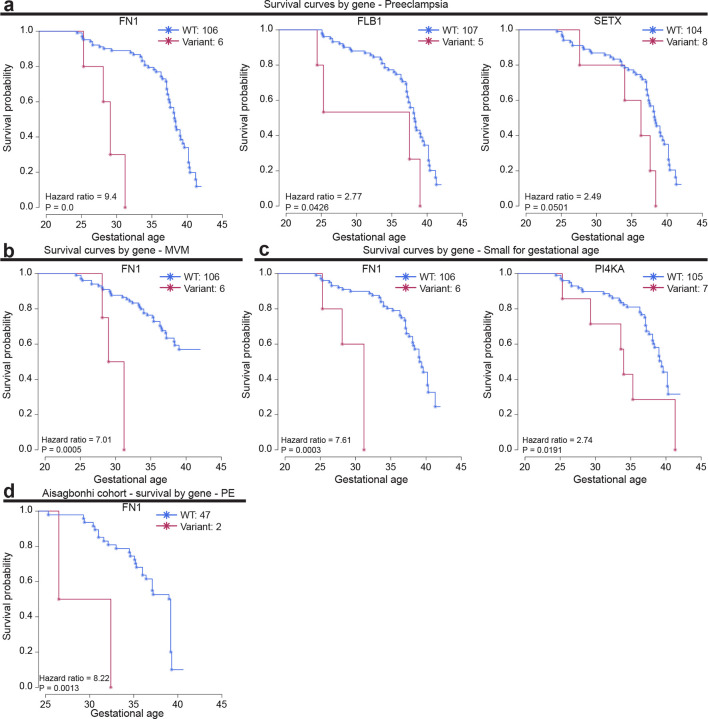
Individual genes harboring rare damaging mutations were associated with increased risk of delivery at earlier gestational ages with preeclampsia, maternal vascular malperfusion, and small for gestational age. **a,** Survival curves by variant in FN1, FLB1, and SETX for preeclampsia. *P* value from Cox proportional-hazards test. **b,** Survival curves by variant in FN1 for maternal vascular malperfusion. *P* value from Cox proportional-hazards test. MVM, maternal vascular malperfusion. **c,** Survival curves by variant in FN1 and PI4KA for small for gestational age. *P* value from Cox proportional-hazards test. **d,** Survival curves by variant in FN1 for preeclampsia in the Aisagbonhi cohort. *P* value from Cox proportional-hazards test. PE, preeclampsia.

Placentas in the discovery cohort with rare damaging variants involved in regulation of transforming growth factor-beta (TGF-β) production (OR: 8.34 (2.05–49.41), P = 0.001), integrin cell surface interactions (OR: 4.34 (1.26–17.35), P = 0.011), extracellular matrix degradation (OR: 3.37 (1.34–8.76), P = 0.005), epithelial cell proliferation (OR: 3.07 (1.21–7.99), P = 0.01), and extracellular matrix organization (OR: 2.67 (1.14–6.38), P = 0.018) had increased risk of delivering early preterm (Fisher’s exact test, Fig. 5c). Placentas harboring rare damaging variants in the cell cycle G1/S phase transition had increased risk of MVM (OR: 3.63 (0.96–15.35), P = 0.033, Fisher’s exact test; Fig. 5c). Within these pathways, individual genes harboring rare damaging mutations were associated with increased risk of delivery at earlier gestational ages with PE, MVM and SGA (P < 0.05, Fisher’s exact test and Cox’s proportional hazards model; Fig. 5d, Extended Data Fig. 3–4). Of these, rare damaging mutations in fibronectin (FN1) were validated in the Aisagbonhi cohort (Cox’s proportional hazards test; Extended Data Fig. 4d). Collectively, these data demonstrate an association between genomic instability and variants that disrupt cellular processes important to placental development and function.

### Functional consequences of genomic instability.

**Fig. 6:**
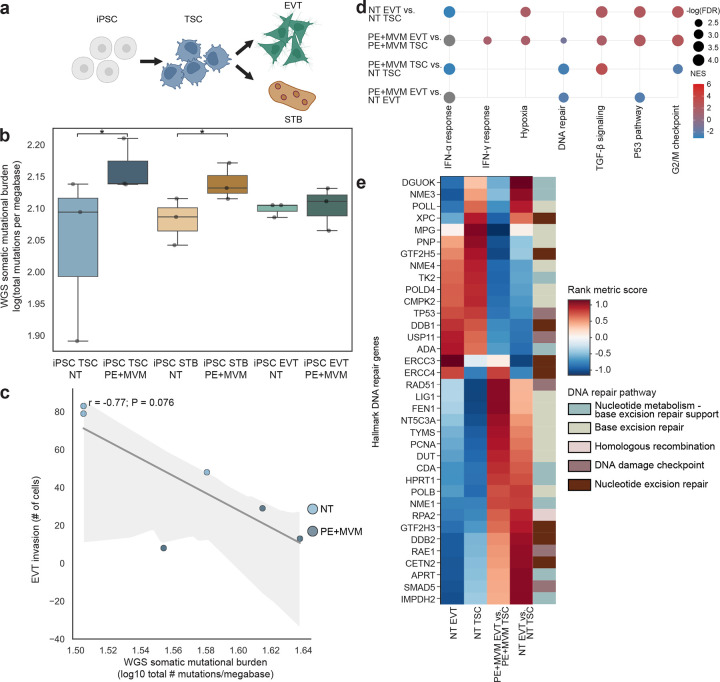
Trophoblast stem cells from preeclampsia and maternal vascular malperfusion placentas demonstrate increased genomic instability and dysregulated DNA repair mechanisms. **a,** Cell line system leveraging reprogrammed umbilical cord tissue derived mesenchymal stem cells reprogrammed to induced pluripotent stem cells converted to trophoblast stem cells and differentiated to extravillous trophoblasts and syncytiotrophoblasts. Created in BioRender. Fisch, K. (2026) https://BioRender.com/1o21983. iPSC, induced pluripotent stem cells; TSC, trophoblast stem cell; EVT, extravillous trophoblast; STB, syncytiotrophoblast. **b,** Somatic mutational burden of cell types stratified by preeclampsia and maternal vascular malperfusion. * *P* < 0.05; *P* value from a one-sided Mann Whitney U-test. WGS, whole genome sequencing; NT, normotensive; PE, preeclampsia; MVM, maternal vascular malperfusion. **c,** Somatic mutational burden of cell types correlated with extravillous trophoblast invasion. *P* value from a Spearman’s rank-order correlation test. **d,** Gene Set Enrichment Analysis of differentially expressed genes from whole transcriptome data of iPSC-TSC and iPSC-EVT reveal significant dysregulation of DNA repair, cell cycle, EMT, hypoxia and interferon response pathways (FDR < 0.05) in cells derived from pregnancies with preeclampsia and maternal vascular malperfusion relative to normotensive without maternal vascular malperfusion. EMT, epithelial mesenchymal transition; FDR, false discovery rate; NES, normalized enrichment score. **e,** Leading edge analysis of the Gene Set Enrichment Analysis rank metric score for genes in Hallmark DNA repair.

We previously developed an *in vitro* model system that recapitulates trophoblast differentiation during both normal development and disease^28^ (Fig. 6a). This cell line model uses umbilical cord mesenchymal stem cell-derived induced pluripotent stem cells (iPSC) that are converted to TSCs (iPSC-TSC), and then further differentiated into extravillous trophoblasts (EVT) or syncytiotrophoblasts (STB), two terminally-differentiated functional cell types in the placenta. Whole-genome sequencing data (30X coverage) from 6 patient-derived iPSC-TSC, iPSC-STB and iPSC-EVT (n = 3 normotensive without MVM; n = 3 PE with MVM) were evaluated for somatic mutations. Gene expression from RNA-seq was evaluated for all six patient samples across the three cell states to identify molecular mechanisms underlying increased genomic instability in PE with MVM by cell type.

We observed cell-type-specific differences in genomic instability, as the genomic instability of PE with MVM TSCs and STBs was higher than that of normal TSCs and STBs (P = 0.05, one-sided Mann-Whitney U test; Fig. 6b, Supplementary Table 13), suggesting a possible mechanism of disease pathogenesis. Interestingly, the number of invasive EVT decreased as genomic instability increased (r = −0.77, P = 0.076, Pearson correlation coefficient; Fig. 6c, Supplementary Table 13), suggesting that changes in genomic instability may disrupt EVT function.

### DNA repair activity in trophoblast stem cells.

Gene Set Enrichment Analysis (GSEA) of differentially expressed genes from RNA-seq of iPSC-TSC and iPSC-EVT revealed dysregulated DNA repair, cell cycle, TGF-β signaling, P53 signaling, hypoxia, and interferon response pathways in cells derived from pregnancies with PE with MVM, relative to those derived from normotensive pregnancies without MVM (False discovery rate < 0.05; Fig. 6d). Leading edge analysis of the GSEA rank metric score for genes in the Hallmark DNA repair pathway revealed dysregulated DNA repair activity in PE with MVM in differentiation of TSCs to EVTs and within both TSCs and EVTs, compared to DNA repair activity in the individual cell types and the normotensive differentiation pathway of TSC to EVT (Fig. 6e, Supplementary Table 14). In normotensive TSC to EVT differentiation, DNA damage checkpoint genes, such as TP53 are activated, along with DNA repair pathways nucleotide excision repair (NER) and base excision repair (BER). Strikingly, within both TSC and EVTs in PE with MVM, we see loss of TP53 expression, along with activation of homologous recombination for double-stranded break repair in TSCs (RAD51 and RPA3)^29,30^, while EVTs with MVM have increased expression of nucleotide metabolism, nucleotide excision repair, and DNA damage checkpoint genes, RAE1 and SMAD5, known to be induced by genotoxic stress^31,32^. Intriguingly, ERCC4 and ERCC1, genes involved in nucleotide excision repair^33^, and genes involved in base excision repair, are significantly downregulated in the PE with MVM context in both EVTs and TSCs and in the differentiation from TSC to EVT in PE with MVM, compared to normotensive (Fig. 6e). These findings suggest a potential mechanism for the observed increase in genomic instability in PE with MVM and cell type-specific induction of DNA repair in EVTs and TSCs resulting from hypoxia and/or genotoxic stress.

## Discussion

In this study, we analyzed bulk RNA-seq of human placentas to determine how genomic instability contributes to maternal and neonatal outcomes, focusing on PE and SGA, as well as on MVM, the placental patterns of injury associated with PE and SGA. We observed an increase in genomic instability in EP-PE placentas (15/20 with MVM, 18/20 with SGA neonates). We found that higher genomic instability indicates an increased risk for PE, MVM, and SGA infants and leads to an increase in the probability of these outcomes at earlier gestational ages. Notably, genomic instability and hypoxia were moderately predictive biomarkers for the presence of PE, MVM, and SGA, all of which were further validated in an independent cohort.

We show that one possible mutagen, oxidative stress, may contribute to the observed genomic instability, as indicated by the presence of the SBSC mutational signature predominant in the EP-PE cohort. The presence of MVM increased the contribution from the oxidative stress (SBSC) mutational signature, which aligns with the fact that MVM causes hypoxia/reoxygenation injury, thereby generating oxidative stress in the placenta. Oxidative stress is a well-established cause of DNA damage^34^, which can lead to genomic instability^35,36^. DNA damage resulting from micro-environmental or endogenous stressors induces activation of DNA damage response pathways^27^. If these response pathways fail to repair DNA damage, somatic mutations can accumulate, leading to altered cellular function or further genomic instability, particularly if the somatic mutations occur in genes involved in DNA repair pathways^27^.

Importantly, genomic instability disrupts pathways important for placental health in all three cohorts analyzed. Placentas in the discovery cohort with rare damaging variants involved in regulation of TGF-β production and epithelial cell proliferation, among others, had increased risk of delivering early preterm. Placentas harboring rare damaging variants in the cell cycle G1/S phase transition had increased risk of MVM. Individual genes in these pathways harboring rare damaging mutations increases risk of delivery at earlier gestational ages with PE, MVM and SGA. Within the placenta, TGF-β plays critical roles in the regulation of trophoblast proliferation and differentiation^37^. Impacted epithelial cell proliferation could be a result of dysregulated TGF-β signaling^38^. TGF-β signaling also regulates EVT invasion and interactions with the maternal uterine environment, facilitating proper vascular remodeling necessary for placental anchoring and nutrient exchange^39^. In PE, however, TGF-β is elevated in the placenta^40^, and its dysregulation is associated with impaired EVT differentiation and invasion, potentially contributing to poor placental function^39^. Genes with rare damaging mutations in TGF-β production, such as FN1, were predictive of poor maternal, placental, and neonatal outcomes – PE, MVM and SGA – with placentas harboring FN1 mutations at higher risk of delivering at lower gestational age, in both the discovery and validation cohorts. These findings suggest that genomic instability may disrupt cellular processes important to placental development and function, and markers of this pathway may be useful for predicting these outcomes.

Interestingly, rare damaging variants and pathways were observed in circulating RNA, suggesting that they have potential to serve as predictive biomarkers for MVM during pregnancy. However, the maternal circulating RNA dataset did not have clinical data for placental histopathology, SGA, or fetal sex. Future studies with matched circulating RNA and placental tissue with detailed placental histopathology and neonatal outcomes are warranted. We also had a limited sample size for maternal outcomes such as EP-PE (n = 20) and we did not have a normotensive SGA group for comparison; thus, future prospective studies including balanced groups and ample sample sizes for each clinical group will increase power. Furthermore, placental variants were filtered for somatic variants only^19^, although some variants identified as somatic could potentially originate from maternal blood contamination rather than placental tissue, warranting study of matched maternal blood and placental tissue to remove artefacts. As such, further studies are needed to elucidate MVM biomarkers in circulating DNA and RNA.

Finally, to test our hypothesis that genomic instability affects placental function, we leveraged an iPSC-derived TSC-based model to evaluate placental cell type-specific findings and to interrogate the impact of genomic instability on altered DNA repair mechanisms. We identified increased genomic instability in PE+MVM iPSC-TSC, iPSC-STB and altered DNA repair mechanisms in PE+MVM TSCs and EVTs. Notably, the observation that increased genomic instability correlated with reduced extravillous trophoblast invasion implicates a functional role for genomic instability. Our findings reveal a striking dysregulation of DNA repair gene expression in PE+MVM-derived trophoblasts. The observed upregulation of homologous recombination genes such as RAD51 and RPA3 in TSCs from PE+MVM pregnancies may reflect a compensatory response to replication stress or hypoxia-induced DNA damage, both of which are common features of the preeclamptic placenta. However, the near-baseline expression of these genes in differentiated EVTs suggests that this response is not sustained across trophoblast lineages, potentially contributing to EVT-specific genomic instability or impaired invasion. The significant downregulation of ERCC1 and ERCC4, key components of nucleotide excision repair, raises the possibility that specific repair pathways are selectively suppressed in PE+MVM, which may compromise the ability of trophoblasts to resolve DNA lesions. Finally, our observation that TP53, an important DNA checkpoint inhibitor that, when lost, contributes to tolerance of genomic instability in cancer^41^, is reduced in both TSCs and EVTs with MVM points to potential defects in DNA repair warranting further study in the context of placental tissue. While these models are derived from reprogrammed umbilical cord-derived mesenchymal stem cells, rather than primary (placenta-derived) TSC, our findings suggest a potential functional aspect to the damaging mutations, in that impaired DNA damage response or increased hypoxia may serve as subclassifications for EP-PE based on the underlying dysregulated mechanisms.

In conclusion, these findings provide promising insights into the underlying mechanisms of genomic instability in the placenta, which may serve as useful biomarkers for early clinical diagnosis of placental injury, such as MVM, underlying PE.

## Methods

### Study cohort

The study design included 112 placenta samples collected from participants at UCSD Health through the UC San Diego Center for Perinatal Discovery Biobank. All biospecimens and clinical data are collected under approval of the Human Research Protections Committee of the UCSD Institutional Review Board (IRB#: 181917X). All samples underwent detailed clinical adjudication and histopathological review independently by two perinatal pathologists to determine the pathology category of PE or normotensive previously described. Early preterm delivery was defined as a gestational age at delivery of less than 34 weeks^16^. PE and SGA were defined following Horii et. al^9^. Clinical demographics violin plots were plotted using seaborn^42^ (v. 0.13.2) in Python (v. 3.12.1). Chi-squared summary statistics to compare cohort demographics were calculated using TableOne (v. 0.9.1).

### RNA-seq library preparation

This unit of work is partially a reanalysis of previously published data^9^, thus, the details of sample preparation are previously described. In brief, total RNA was isolated from placental villous tissue using mirVana RNA Isolation Kit (ThermoFisher). RNA concentration was measured using Qubit RNA BR Assay Kit (ThermoFisher). RNA integrity was checked using RNA 6000 Nano chip read by Agilent 2100 bioanalyzer. All samples had an RNA integrity number above 7.0. RNA-seq libraries were sequenced on a NovaSeq6000 System (Illumina) through the UCSD Institute for Genomic Medicine Genomics Center.

### RNA-seq somatic variant calling

Quality control was performed using FastQC^43^ (v. 0.11.3). The bcbio-nextgen^44^ (v. 1.2.9) Bulk RNA-seq workflow was used for alignment to the human genome hg38 with STAR^45^ (v. 2.6.1d)-2 Pass Genome alignment and variants calling using the GATK Best Practices Workflow for RNA-seq short variant discovery^46^ (v. 4.2.2.0). Using bcftools^47^ (v. 1.17), the following post-processing filters were applied to the variant calls: DP>10, GQ>20, QD>5, MQ>40, ReadPosRankSum>−3, MQRankSum>−10, FS<200, and QUAL>50 and possible RNA editing sites marked by bcbio when available. To filter for somatic variants, we followed the protocol from Eberth et. al^19^ (v. 1.0.0). In brief, we further filtered out RNA editing sites previously identified by REDIPortal^48^ (v 3.0), low complexity regions, and common variants. Ensembl Variant Effect Predictor^49^ (v. 107) vcf2maf^50^ (v. 1.6.21) were then used for variant annotation.

### Mutational signatures

MutationalPatterns^51^ (v. 3.14.0) and non-negative matrix factorization (NMF, v.0.28) was used to characterize mutational signatures on the variant call format files. Heatmaps of mutational signature were plotted using the plot_contribution_heatmap function. Spearman rank-correlation test was used to correlate mutational signatures to genomic instability using lmplot in seaborn. Spearman correlation coefficient and associated p-value were calculated using the spearmanr function from scipy.stats^52^ (v. 1.13.1).

### Rare, damaging variant analysis

We filtered variants passing quality control for variants with a coverage depth > 50. To identify rare variants, we retained variants with a maximum observed allele frequency in 1000 Genomes (MAX_AF) of < 0.01 which was implemented using maftools^53^ (v. 2.22.0). Additionally, variants that were present in more than 70% of samples or if the gene with the variant is present in more than 50% of samples were filtered out. We identified rare, damaging variants as rare variants that are predicted to damage protein product by filtering for mutations designated as “damaging” by PolyPhen-2^54^, “deleterious” by SIFT^55^, removing any mutations designated as “benign” by ClinVar^56^ CLIN_SIG and retaining rare nonsense and nonstop mutations. The validation and circulating cohorts underwent the same filtering for rare damaging variants, but with a lower coverage depth > 10 to validate those identified in the discovery cohort.

### Calculating genomic instability

Genomic instability was defined as the number of expressed rare variants per capture region (50x coverage for placental RNA, 10x coverage for circulating RNA) per 10^6 bases based on expressed mutational burden^20^. Genome coverage of each sample was determined using bedtools (v. 2.30.0) genomeCoverageBed.

### Differential expression analysis

The bcbio-nextgen Bulk RNA-seq workflow performed transcript quantification with salmon^57^ and calculated transcript read abundance using tximport^58^. The R (v 4.1.3) Bioconductor package tximport (v.1.22.0) was used to import per-sample transcript read abundance (lengthScaledTPM) and aggregate it at the gene level prior to TMM-normalization and differential expression analysis using the Bioconductor packages edgeR^59^ (v.3.26.0) and limma^60^ (v. 3.50.3) to implement the limma-voom method^61^. Genes were considered significantly differentially expressed after adjustment for multiple testing using the moderated t-statistic (adjusted p-val < 0.05) and an abs(log2 fold change) >1. g:Profiler^62^ was used for gene set enrichment analysis with the differentially expressed genes as input and a custom background of all expressed genes. Pathways were considered significant at an adjusted p-value < 0.05 to account for multiple comparisons.

### Hypoxia gene scoring

We followed the gene scoring method of Bhandari et al., 2020^63^. The median mRNA expression level across our bulk RNA-seq samples was computed for every gene for hypoxia gene signatures^24^. Samples with mRNA expression levels greater than the gene’s associated median were assigned a score of +1 and those with expression levels below the median a score of −1. The sum of these individual scores for each sample across the three signatures in a given gene was the resulting gene’s score.

### Statistical analyses

Boxplots were plotted using seaborn^42^ (v. 0.11.2). We utilized two-sided Mann-Whitney U tests with Benjamini-Hochberg correction to compare mean ranks unless otherwise noted. One-sided tests were performed based on the hypothesis being tested, which is stated accordingly. We performed Pearson correlation to uncover the relationship between EVT invasion (number of cells) and mutational burden.

### Predictive modeling

Cox proportional hazard model was built using the CoxPHFitter function from lifelines^64^ (v. 0.30.0) using genomic instability, hypoxia score, maternal age, and fetal sex as features. First, we used the k_fold_cross_validation function to identify an appropriate penalizer by comparing log-likelihood. We then verified the Cox proportional hazard model and penalizer passed all proportional hazard assumptions using the check_assumptions function with a p-value threshold of 0.05. Variables that failed the non-proportional test were squared to change their functional form, following the lifelines^64^ workflow. We plotted the Cox proportional hazard model using the plot_partial_effects_on_outcome function with the 10th and 90th percentile of the feature of interest.

Feature selection was performed using five-fold cross-validation in scikit-learn^65^ (v. 1.6.1) on the discovery cohort. This was done to evaluate model accuracy and determine whether maternal age and fetal sex should be included as features alongside genomic instability and hypoxia gene scoring. Based on this analysis, the KNeighborsClassifier was selected and subsequently used with default parameters for all predictive models. The validation cohort was used exclusively for testing. Receiver operating characteristic curves and AUC values were calculated using the ROCAUC function from Yellowbrick^66^ (v. 1.5).

### Validation and circulating cohort

The data for the Aisagbonhi cohort was downloaded from Gene Expression Omnibus (GSE234729)^27^. The data for the Moufarrej cohort was downloaded from Sequence Read Archive (SRP352519)^26^. The data for the trophoblast stem cells was downloaded from Gene Expression Omnibus (GSE243579)^67^. These datasets were all subject to the variant calling and analysis.

### iPSC trophoblast cell line transcriptome analysis.

We obtained published EVT invasion data and raw counts from RNA-seq data from GSE243579 for N=6 iPSC-derived trophoblast stem cells and their differentiated derivatives (N=36 RNAseq samples across two cell types in triplicate (N=18 samples TSC, N=18 samples EVT, from N=6 iPSC patient lines). RNA-seq data was analyzed as described above. In addition, we performed Gene Set Enrichment Analysis (GSEA v. 4.4.0) on MSigDB Hallmark pathways to identify activated pathways in normotensive-MVM TSC to EVT differentiation and by cell type within TSC and EVT in PE+MVM relative to normotensive-MVM lines.

### iPSC trophoblast cell line whole genome sequencing analysis

We obtained extracted DNA from all six cell lines from Dr. Horii’s laboratory. Whole genome sequencing (WGS) was performed at 30x coverage on a NovaSeqX System (Illumina) at the UCSD IGM Genomics Center. WGS was first aligned using bwa mem (v. 0.7.17) with the GRCh38.d1.vd1 Reference Sequence from the National Cancer Institute’s Genomic Data Commons^68^. Variants were then called using the GATK (v 4.2.6.1) Mutect2 workflow for single sample somatic variant calling following the “(How to) Call somatic mutations using GATK4 Mutect2” tutorial^46^. Ensembl Variant Effect Predictor^49^ (v. 107) and vcf2maf^50^ (v. 1.6.21) were then used for variant annotation using GRCh38. We filtered for variants passing quality files and with a depth > 30 using maftools^53^ (v. 2.22.0). Then, we removed any variants that were observed in > 33% of samples^69^.

## Extended Data

## Supplementary Files

Supplementary Table 1. Chi-squared statistics for metadata stratified by normotensive and preeclampsia.

Supplementary Table 2. Chi-squared statistics for metadata stratified by non-maternal vascular malperfusion and maternal vascular malperfusion.

Supplementary Table 3. Rare variants and their associated variant annotations in the discovery cohort.

Supplementary Table 4. Rare variants and their associated variant annotations in the Aisagbonhi cohort.

Supplementary Table 5. Rare variants and their associated variant annotations in the Moufarrej cohort.

Supplementary Table 6. Differential expression between early preterm delivery with preeclampsia and near-term/term delivery normotensive.

Supplementary Table 7. Differential expression between near-term/term delivery with preeclampsia and near-term/term delivery normotensive.

Supplementary Table 8. Differential expression between near-term/term delivery with preeclampsia and early preterm delivery with preeclampsia.

Supplementary Table 9. Differential expression between near-term/term delivery normotensive and early preterm delivery normotensive.

Supplementary Table 10. Differential expression between early preterm delivery with abnormal pathology and near-term/term delivery with normal pathology.

Supplementary Table 11. Enrichment for genes with variants in the discovery cohort.

Supplementary Table 12. Enrichment for genes with variants in the circulating cohort.

Supplementary Table 13. Metadata of trophoblast stem cell lines partially derived from GSE243579^67^.

Supplementary Table 14/ Pathways of variants in extravillous trophoblasts and trophoblast stem cells between normotensive without maternal vascular malperfusion and preeclampsia with maternal vascular malperfusion.

## Data Availability

Raw and processed sequencing data from 76 placentas generated in this study are available from Sequence Read Archive under accession SRP466001 and BioProject under accession PRJNA1027377. Raw and processed sequencing data from 36 placentas were a reanalysis of the data generated in Horii et. al^9^, and are available from the Gene Expression Omnibus database under the accession number GSE186257. Raw data from the Aisagbonhi cohort and the Moufarrej circulating cohort were obtained from GSE234729 and SRP352519.

## Code Availability

Analysis code is available on GitHub: https://github.com/UCSD-Fisch-Lab/placenta_genomic_instability.
